# Actin polymerization counteracts prewetting of N-WASP on supported lipid bilayers

**DOI:** 10.1073/pnas.2407497121

**Published:** 2024-12-04

**Authors:** Tina Wiegand, Jinghui Liu, Lutz Vogeley, Isabel LuValle-Burke, Jan Geisler, Anatol W. Fritsch, Anthony A. Hyman, Stephan W. Grill

**Affiliations:** ^a^Max Planck Institute of Molecular Cell Biology and Genetics, Dresden 01307, Germany; ^b^Max Planck Institute for the Physics of Complex Systems, Dresden 01187, Germany; ^c^Center for Systems Biology Dresden, Dresden 01307, Germany; ^d^Max Planck School Matter to Life, Heidelberg 69120, Germany; ^e^Cluster of Excellence Physics of Life, Technische Universität Dresden, Dresden 01307, Germany

**Keywords:** cortical condensates, prewetting, in vitro actin cortices

## Abstract

Cortical condensates, transient punctate-like structures rich in actin and the actin nucleation pathway member Neural Wiskott-Aldrich syndrome protein (N-WASP), form during activation of the actin cortex in the *Caenorhabditis elegans* oocyte. Their emergence and spontaneous dissolution is linked to a phase separation process driven by chemical kinetics. However, the mechanisms that drive the onset of cortical condensate formation near membranes remain unexplored. Here, using a reconstituted phase separation assay of cortical condensate proteins, we demonstrate that the key component, N-WASP, can collectively undergo surface condensation on supported lipid bilayers via a prewetting transition. Actin partitions into the condensates, where it polymerizes and counteracts the N-WASP prewetting transition. Taken together, the dynamics of condensate-assisted cortex formation appear to be controlled by a balance between surface-assisted condensate formation and polymer-driven condensate dissolution. This opens perspectives for understanding how the formation of complex intracellular structures is affected and controlled by phase separation.

## Significance

Liquid–liquid phase separation has emerged as a key mechanism for intracellular compartmentalization. How proteins condense in cells under nonequilibrium conditions remains a fundamental question. While chemical reactions can drive the formation and dissolution of biomolecular condensates, the onset of the phase separation process is less well characterized. Here, we pursue a reconstitution approach to show that Neural Wiskott-Aldrich syndrome protein (N-WASP), a central protein component of cortical condensates, is capable of undergoing a prewetting transition to form surface condensates on supported lipid bilayers. We also find that actin polymerization counteracts N-WASP prewetting. Our work provides perspectives for understanding how phase separation can shape the intracellular environment.

Surface transitions of biomolecules near membranes can serve as an important mechanism to localize biochemical reactions in the cell. The affinity of proteins to membranes can induce their phase separation on the membrane (1–4), far below the saturation concentration for bulk liquid–liquid phase separation (LLPS) (5, 6). Condensation at surfaces below the saturation concentration can arise through prewetting, a process related to wetting phenomena (7). The prewetting transition describes the abrupt switch of a single-molecule-thin layer of molecules adsorbed on a surface toward a thick layer (8). In the context of biological systems, prewetting transitions have for example been reported for sequence-dependent protein condensation processes on DNA (9). For biological membranes, prewetting transitions play a role in protein condensation on the lipid surface (10–14). However, how prewetting condensates form on membranes remains poorly understood. Furthermore, understanding wetting phenomena in biological systems that typically are far from equilibrium remains challenging. While the principles of LLPS are still applicable within localized regions within cells that remain equilibrated (15), chemical reactions can drive condensates out of equilibrium and significantly influence phase separation kinetics (16–18). Condensates can be dynamic in nature, and recruit additional components (19) that drive specific biochemical reactions, such as actin polymerization (2, 20–22), which can even lead to their dissolution (23). Examples for dynamic condensates forming in vivo are the actin fusion focus in budding yeast (24), and cortical condensates that transiently form prior to their self-organized disassembly at the oocyte to embryo transition (25).

These two examples indicate that condensates that form in the cytoskeletal context can be highly dynamic. Indeed, the activation of the actomyosin cortex in the *Caenorhabditis elegans* embryo is accompanied by the transient formation of hundreds of cortical condensates that are rich in the actin nucleation pathway member Neural Wiskott-Aldrich Syndrome Protein (N-WASP), the branched nucleator Arp2/3, and actin. Cortical condensates exhibit unconventional chemical dynamics and a condensate dynamic instability that orchestrates a switch from condensate growth to shrinkage and dissolution (25). The molecular mechanisms that are at the heart of this collective switch remain unclear. Furthermore, N-WASP has been shown to undergo LLPS in vitro together with its adapter proteins NCK (non-catalytic region of tyrosine kinase adaptor protein 1) and Nephrin, but it remains unclear how this behavior impacts on cortical condensate formation (26, 27).

Here, we set out to understand the mechanisms that drive the condensation of cortical condensates and that orchestrate their switch to disassembly. We employ an in vitro approach to investigate the formation of liquid-like condensates on supported lipid bilayers (SLBs) in order to reveal the spatiotemporal dynamics of key proteins that constitute cortical condensates dynamics. Our study encompasses two variants of N-WASP, human and *C. elegans*, in order to allow for comparisons with previously published findings, both in human and *C. elegans* systems.

## Results

### N-WASP Condenses in Absence of Adaptors In Vitro.

**Fig. 1.**
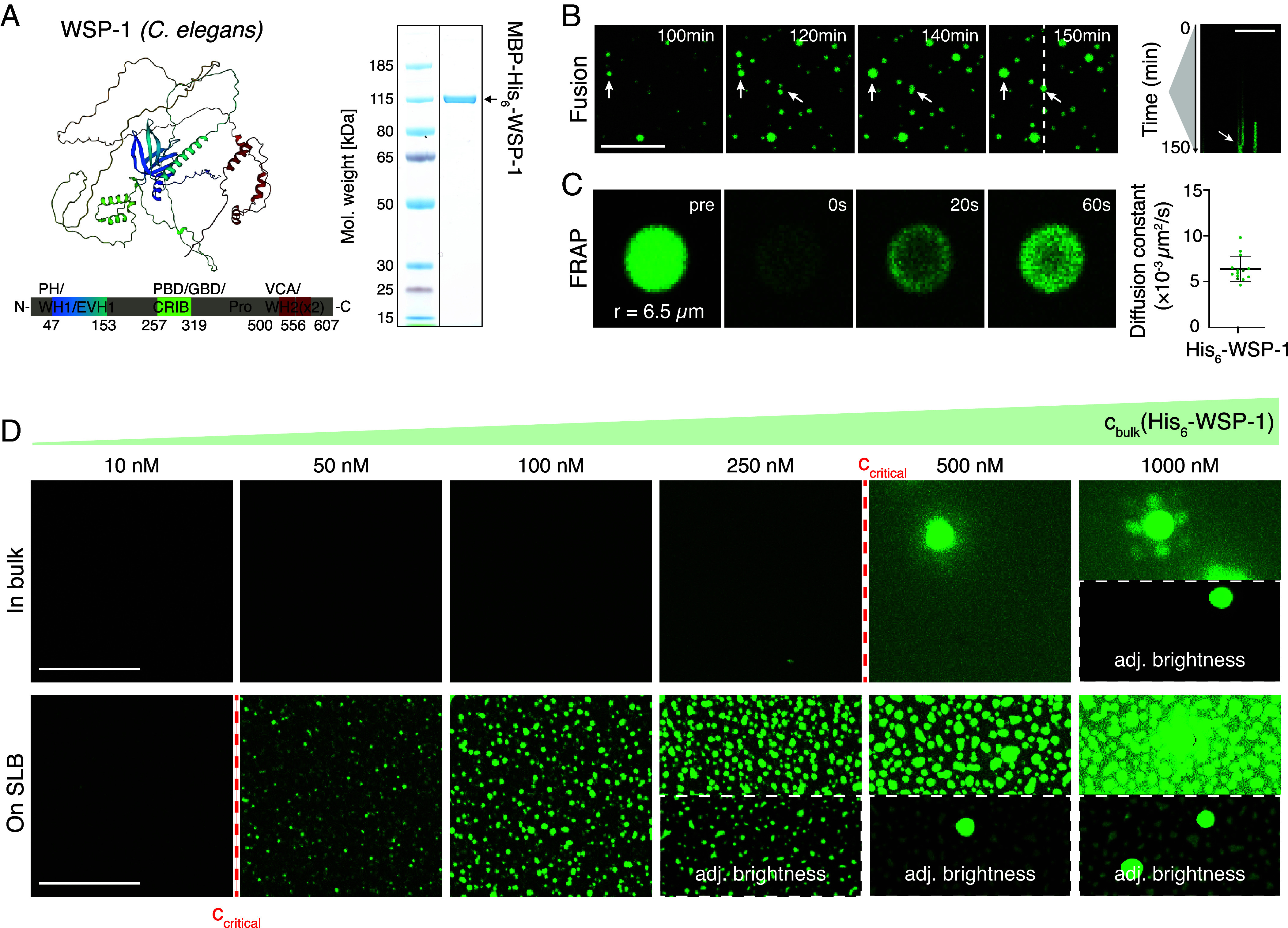
N-WASP undergoes phase separation without adapter proteins in vitro. (*A*) Alpha fold predicts large disordered regions for *C. elegans* N-WASP (WSP-1) (sequence-dependent color code). Sodium dodecyl sulfate gel shows recombinantly expressed and purified MBP-His_6_-WSP-1 (for activity controls and results of human N-WASP see *SI Appendix*). (*B*) Confocal time-lapse of His_6_-WSP-1 (5 μM, 10% 488-tagged, MBP-tag cleaved right before experiment; see *SI Appendix*) forming droplets in bulk upon lowering KCl concentration to 150 mM. Kymograph indicates growth of droplets and fusion (white arrows). (*C*) Fluorescence recovery after photobleaching (FRAP) images of condensate formed from 5 μM His_6_-WSP-1 between 5 to 60 min. Diffusion coefficients D inside the condensates were determined for n = 14 condensates. Data are the mean ± SD. (*D*) Phase separation assays of His_6_-WSP-1 reveal a critical concentration for phase separation of 500 nM in bulk (*Upper* row; see *SI Appendix*, Fig. S1*E*) and 50 nM on a supported lipid bilayer (SLB) with 1% Ni-NTA lipids (*Lower* row). (All scale bars: 10 μm.)

In the *C. elegans* oocyte, typical N-WASP interaction proteins like NCK-1 or Cdc42 (Cell division control protein 42 homolog) are dispensable for the formation of cortical condensates (25). We first asked whether homotypic interactions between N-WASP proteins are sufficient to drive N-WASP condensation. We purified full-length N-WASP, both from human and *C. elegans*, recombinantly and performed phase separation assays in vitro (for quality controls of different protein constructs and activity assay toward actin polymerization see Fig. 1*A* and *SI Appendix*, Fig. S1 *A*–*C*). We find that purified full-length *C. elegans* N-WASP undergoes phase separation at physiological salt concentrations (150 mM KCl) in vitro without further adaptor proteins or crowding agents (Fig. 1*D* and *SI Appendix*, Fig. S1*D*). The critical concentration for phase separation in bulk solution is 947 ± 621 nM, as determined by the volume fraction of the condensed phase in bulk (*SI Appendix*, Fig. S1*E*). However, typical concentrations of N-WASP in *C. elegans* embryos are ∼100 nM (28), indicating that bulk phase separation of N-WASP should not take place in vivo. We next focused on human N-WASP (*SI Appendix*, Fig. S2 *A* and B), which similarly undergoes LLPS without additional proteins. We determined the associated saturation concentration to be 2.17 ± 0.44 μM (*SI Appendix*, Fig. S2*C*). We conclude that homotypic interactions are sufficient to drive bulk condensation of N-WASP.

### N-WASP In Vitro Condensates Show Liquid Features.

In vivo cortical condensates were found to form via a mechanism different from equilibrium LLPS, but display characteristics indicative of rapid internal mixing (25). Do in vitro N-WASP condensates also show liquid-like features? We performed time-lapse imaging and observed growth and fusion (Fig. 1*B* and Movie S1) of in vitro condensates of *C. elegans* N-WASP. We further conducted FRAP experiments showing exchange of molecules between the condensates and the surrounding dilute phase (Fig. 1*C*) and determined the diffusion rate within the condensates to be 0.006 ± 0.001 μm^2^/s (mean ± SD, Fig. 1*D*). Similarly, human N-WASP in vitro condensates also exchange material with the dilute phase. Compared to *C. elegans* N-WASP, we observed the diffusion coefficient of human N-WASP inside the condensates to be higher (0.098 ± 0.036 μm^2^/s), likely a consequence of differences in protein length and structure (*SI Appendix*, Fig. S2*D*). These diffusion coefficients are within reported ranges for reconstituted protein condensates (29). We conclude that N-WASP condensates show liquid features such as coalescence, fusion, and diffusion.

### N-WASP Forms Condensates on SLBs at Physiological Concentration.

Typical in vivo concentrations of N-WASP are below the critical concentration (c_sat_) of bulk condensation of N-WASP (*SI Appendix*, Fig. S1*E*). Do interactions with membranes allow for N-WASP condensation below c_sat_? To answer this question, we performed phase separation assays in the presence of SLBs. We found that SLBs containing PIP2 (Phosphatidylinositol 4,5-bisphosphate) or Cdc42 (30, 31) could recruit N-WASP to form small surface puncta not displaying liquid features (*SI Appendix*, Fig. S17 and Movie S8). It is possible that PIP2 and Cdc42 need to act cooperatively to enable N-WASP to form surface condensates resembling in vivo conditions (30–33). To achieve surface condensation, we utilized His-tagged N-WASP, adjusting the surface interaction strength by tuning the concentration of DGS-Ni-NTA within the SLB. At physiological N-WASP concentration (100 nM), dense objects appear to form on SLBs at 0.5% DGS-Ni-NTA (*SI Appendix*, Fig. S3*A*). We used 1% DGS-Ni-NTA for subsequent experiments, as the condensates resemble in vivo appearance and show liquid features (*SI Appendix*, Fig. S3*B* and Movie S2). At 1% DGS-Ni-NTA content condensates start forming on the membrane at bulk His-N-WASP concentrations of 50 nM (Fig. 1*D*, *Lower* row and *SI Appendix*, Fig. S3*D*). We asked whether these objects are monolayered or multilayered structures. If they were monolayered, their integrated fluorescence intensity should scale linearly with covered area on the SLB. If they were multilayered without uniform thickness, the integrated N-WASP fluorescence intensity should scale with covered area with an exponent greater than one (*SI Appendix*). We find that at 100 nM bulk concentration, this scaling exponent is above one: 1.10±0.06 for *C. elegans* and 1.12±0.04 for human N-WASP (*SI Appendix*, Figs. S3*C* and S4, also see *SI Appendix*, section on multilayered condensates). We conclude that N-WASP can form surface condensates that are multilayered and of nonuniform thickness. These form at concentrations significantly lower than the critical concentration c_sat_ for bulk phase separation.

### Abrupt N-WASP Surface Condensation Following N-WASP Adsorption.

**Fig. 2.**
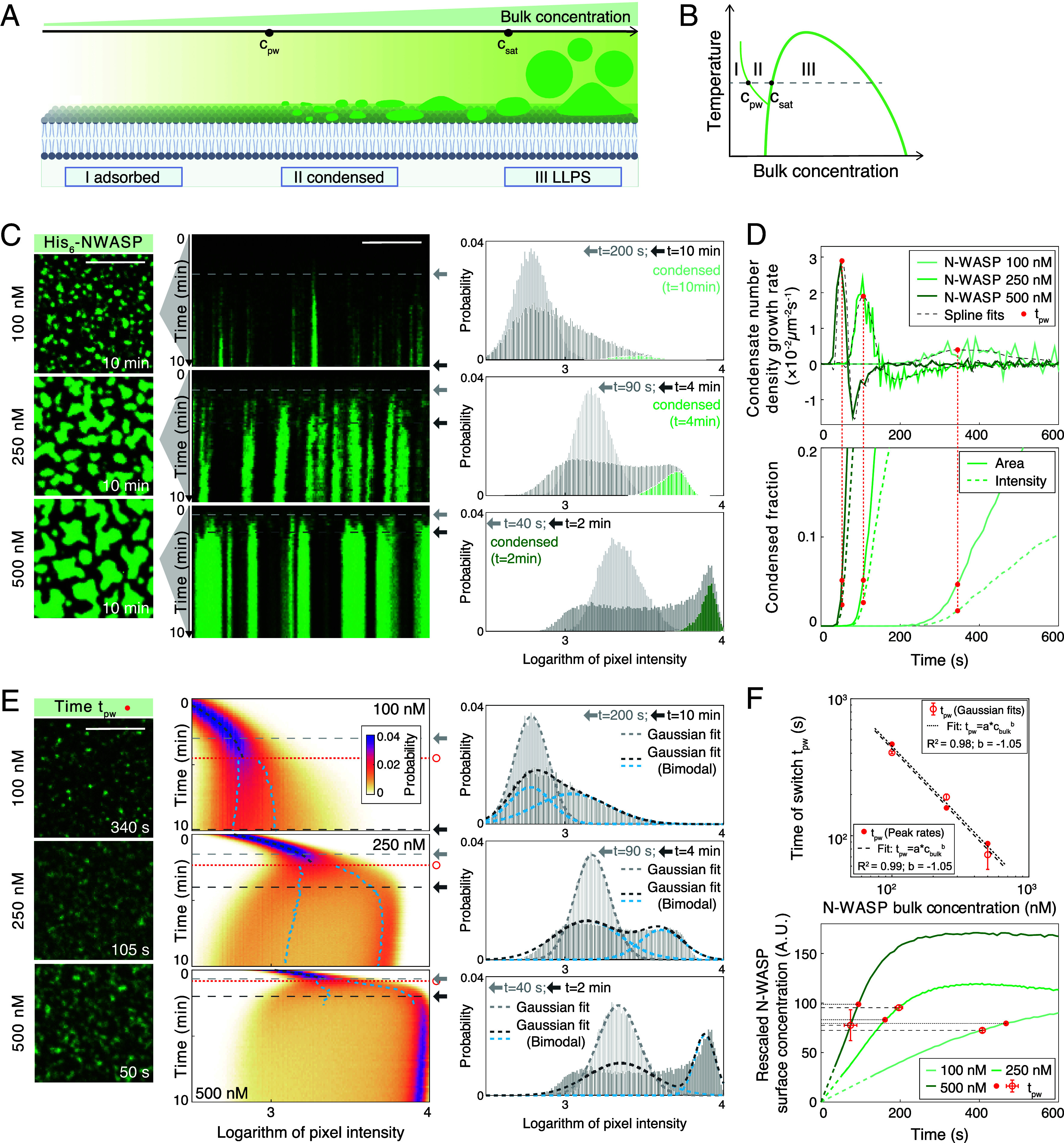
N-WASP prewets on supported lipid bilayers. (*A*) Protein interactions with membranes depend on their bulk concentration. Three protein phases exist: a dissolved phase in the bulk, an adsorbed phase on the SLB, and a condensed phase on the SLB. At low concentrations, proteins adsorb as a thin layer (I, *Left*). Crossing the critical concentration for prewetting (c_*pw*_) condensed areas form (II, *Middle*). Above the saturation concentration (c_*sat*_) for liquid–liquid phase separation (III, LLPS) droplets form spontaneously in bulk (*Right*). (*B*) Phase diagram depicting these three regions of a binary fluid in the presence of a surface. Prewetting line discriminates between areas of adsorption and surface condensation (prewetting). (*C*) Confocal images of human His_6_-N-WASP (10% AF488 labeled, 100 nM *Upper* row, 250 nM *Middle*, and 500 nM *Lower*) condensates on SLBs after t = 10 min. (Scale bar: 10 μm.) Kymographs show adsorption and condensation phase over 10 min (Movie S5). Histogram of the logarithm of pixel intensities (*Right*) shows enrichment of the condensed phase over time. Two distinct time points marked with light gray and dark gray arrows are chosen respectively for each time series. (*D*) Condensate number density growth rate (*Upper*) and condensed phase area (*Lower*, solid lines) and intensity (*Lower*, dashed lines) fractions quantified as a function of time from (*C*). Red filled circles show the time when maximum condensation rate is reached. (*E*) *Left*: Confocal images at the time point (red filled circles) identified in (*D*). *Middle*: Probability density kymographs of the logarithm of pixel intensities for time-lapse images from (*C*). Gray lines (single Gaussian) and blue lines (sum-of-two-Gaussians) represent overlays of probability density fits from either method. The time point of high- and low-intensity-branch splitting (red open circles) is determined by comparing fitting residues between the two methods. *Right*: Histograms and fits (gray dashed lines) for the 3 different N-WASP bulk concentrations each at 2 distinct time points (arrows). In the case of a sum-of-two-Gaussians fit, blue dashed lines show respectively the fitted lower and higher Gaussian peaks. (*F*) *Upper*: The time of abrupt switch inversely scales with initial bulk concentrations. Error bars represent the range extracted from 10% variation of the threshold used for determining $t_{\mathrm{pw}}$. For segmented images, the threshold is the growth rate of condensate number density; For splits of probability density branches, the threshold is the residue difference between single- and sum-of-two Gaussian fits (*SI Appendix*). *Lower*: The curated (*SI Appendix*) mean pixel intensity at the time of abrupt switch is comparable across different initial bulk concentrations.

Our results indicate that N-WASP at physiological concentrations can form condensates on surfaces. Next, we sought to understand the mechanism by which N-WASP forms such multilayered surface-associated condensates. Above the critical saturation concentration for LLPS, condensates formed in bulk can wet biological surfaces (Fig. 2 *A* and *B*, III). Affinity-induced adsorption leads to proteins having increased concentrations near biological surfaces (Fig. 2 *A* and *B*, I). Hence, surface condensates can form below the saturation concentration via a prewetting transition (Fig. 2 *A* and *B*, II). In our in vitro experiments, do N-WASP molecules undergo a prewetting transition on the supported lipid bilayer surface? The prewetting transition is discontinuous, and the height of the surface layer should increase abruptly as bulk concentration increases beyond the prewetting concentration (7). We anticipate to observe signatures of this critical behavior at the onset of in vitro cortical condensate formation, as seen for example for prewetting condensate formation of transcription factors on DNA (9). There, histograms of surface protein amounts underwent a split up with time post adsorption of proteins to the DNA surface, into an absorbed layer with low protein amounts and a condensed layer with higher protein amounts.

To check for signatures of a prewetting transition for our in vitro cortical condensates, we set out to examine the kinetics of N-WASP accumulation on supported lipid bilayers (Fig. 2*C* and *SI Appendix*, Fig. S7 *A* and *B*). We focused on the human N-WASP because of its higher mobility in bulk condensates and its reduced propensity of hardening (*SI Appendix*, Fig. S2*D*, compared to the *C. elegans* variant Fig. 1*C*). At time t=0, we exposed below-saturation ($c_{0}<c_{\text{sat}}$) concentrations of N-WASP solutions to supported lipid bilayers (1% Ni-NTA), and recorded time series of N-WASP intensities. Fig. 2*C* shows the fluorescent images at t=10 min and the corresponding kymographs of N-WASP intensities for 3 representative N-WASP solution concentrations (100 nM, 250 nM, and 500 nM). At the beginning of experiments, we observed continuous association of N-WASP molecules onto the lipid bilayers. The mean pixel intensity appears to follow first-order surface binding kinetics, where the rate of surface association is proportional to the N-WASP bulk concentration (*SI Appendix*, Figs. S7*D* and S8 *A* and *B*). During the initial association process, the probability density histogram of pixel intensities shows a unimodal distribution at low intensities (Fig. 2*C*), consistent with N-WASP molecules individually adsorbing onto the lipid bilayer from the dissolved phase in bulk (9). At a later time, N-WASP condensates appear on the lipid bilayer. Correspondingly, the probability density histogram of pixel intensities switches to a bimodal distribution. This indicates two modes of surface association from the bulk phase: individually associated N-WASP molecules in an adsorbed phase (corresponding to the lower-intensity peak in the histogram; Fig. 2*C*, *Right* panel), and collectively associated N-WASP molecules in a condensed phase (corresponding to the high-intensity peak in the histogram; Fig. 2*C*, *Right* panel). By analyzing this intensity histogram as a function of time, we find that the transition from unimodal to bimodal distribution takes the form of an abrupt switch: The peak representing the adsorbed phase decreases in amplitude, while a second peak rapidly emerges and shifts to higher intensities (Fig. 2*E*; see *Materials and Methods*). We observe a similar abrupt switch for *C. elegans* N-WASP condensation on SLB when using histidine-NTA chemistry (*SI Appendix*, Fig. S18). The switch is absent when *C. elegans* N-WASP is recruited to the SLB via Cdc42 (*SI Appendix*, Fig. S18). Both variants of N-WASP condensates show layer indices that are consistently above one when using histidine-NTA chemistry (*SI Appendix*, Figs. S3*C* and S4*D*), suggesting that they are multilayered surface objects in contact with the adsorbed layer. In addition, photobleaching experiments revealed that N-WASP molecules at the SLB exchange between condensed, adsorbed, and bulk phases as fluorescence recovery is dependent but not sufficiently explained by lateral diffusion (*SI Appendix*, Fig. S9). Altogether, our results are consistent with the condensed phase of N-WASP on the surface forming via an abrupt switch-like transition from the adsorbed layer.

### Evidence for N-WASP Surface Condensates Forming via a Prewetting Transition on the Lipid Bilayer.

The theory of prewetting predicts that surface condensates form upon surpassing of a common critical surface concentration (7). Therefore, we set out to determine whether such a critical concentration exists in our in vitro N-WASP condensation experiments. To obtain microscope images with an acceptable signal-to-noise ratio, these were recorded under conditions that varied with the experimental condition, thus precluding a direct comparison of intensities. We developed two approaches to reliably determine a characteristic time for the onset of condensation directly from imaging data. First, we determined the time at which the rate of N-WASP condensate formation peaks, by segmenting N-WASP condensates and measuring the condensation rate as a function of time (Fig. 2*D*, *Upper* and *SI Appendix*, Fig. S10 *A* and *B*). Second, we determined the time at which N-WASP intensities no longer form a single peak characteristic of the dilute phase, by fitting multivariate Gaussian functions to the time-dependent histogram of pixel intensities (Fig. 2*E*, *Right* and *SI Appendix*, Fig. S11 *A*–*C* and *Materials and Methods*). We find that with both approaches the characteristic time of condensation onset appears to scale inversely with bulk concentration, with a close to −1 exponent, $t_{\mathrm{pw}}\propto c_{\text{bulk}}^{-1}$ (Fig. 2*F*, *Upper* and *SI Appendix*, Fig. S11*D*). This scaling coefficient is consistent with first-order adsorption kinetics. Importantly, this provides evidence that the onset of N-WASP condensation occurs at a common surface concentration. This statement is corroborated by the finding that correspondingly normalized N-WASP fluorescence intensities, which are a measure of surface concentration, are of comparable values at this time of onset of condensation (Fig. 2*F*, *Lower* and *SI Appendix*). Taken together, our data are consistent with a scenario where N-WASP condenses whenever the N-WASP surface concentration on the bilayer surpasses a critical value. This is indicative of N-WASP condensates forming on the lipid bilayers via prewetting transition.

### Actin Limits Final Size of In Vitro N-WASP Condensates.

**Fig. 3.**
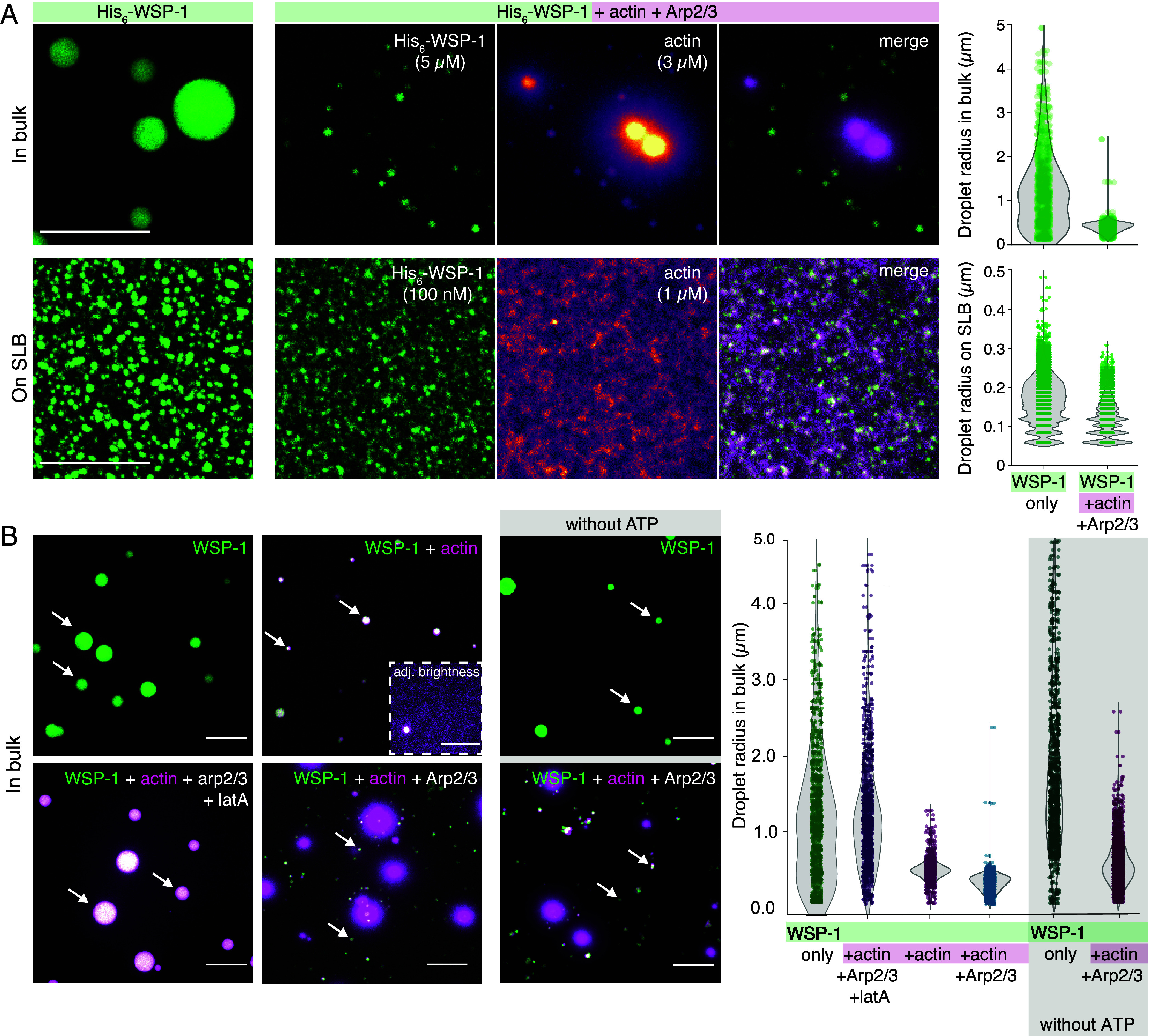
Actin controls equilibrium size of N-WASP condensates in vitro. (*A*) *Left*: Maximum intensity projections of confocal z-stacks of His_6_-WSP-1 (5 μM, 10% AF488-labeled) condensates formed over 1 h in bulk (*Upper* row; see Movie S3) and on a SLB (*Lower* row; see Movie S4) in actin polymerization buffer alone (*Left* images), and in the presence of actin (10% AF647 labeled, magenta) and Arp2/3. *Right*: Quantification of N-WASP droplet radii from max projections. (*B*) *Left*: Maximum intensity projections of confocal z-stacks of His_6_-WSP-1 (5 μM, 10% AF488-labeled) condensates formed over 1 h in bulk alone, or in the presence of actin (3 μM, 10% AF647 labeled, magenta), actin and Arp2/3 (100 nM), and in the presence of latrunculin A (50 μM). For conditions without ATP (*Right* images), the nucleotide on actin was exchanged with AMP-PNP. Arrows point to example N-WASP bulk condensates. *Right*: Quantification of bulk condensate radii from maximum intensity projections. Note that lanes 1 and 4 include the data shown in panel (*A*), *Top* row. (All scale bars: 10 μM.)

In the *C. elegans* embryo, F-actin accumulates in cortical N-WASP condensates over time. This results in negative feedback onto N-WASP and a dynamic instability via a self-organized switch to cortical condensate disassembly (25). Do in vitro N-WASP condensates nucleate the growth of actin fibers, and do these then limit N-WASP condensate growth? We set out to answer this question separately in bulk and on SLBs. First, we added actin and Arp2/3 during the formation of bulk *C. elegans* N-WASP condensates under polymerizing conditions [buffer contained 0.1 mM Mg^2+^ and 0.2 mM Adenosine triphosphate (ATP)]. We find that actin monomers concentrate in N-WASP condensates and that actin fibers polymerize and grow out of the condensates (Fig. 3*A* and Movie S3). Notably, at later times some condensates become smaller (Movie S4 and *SI Appendix*, Fig. S5*A*) and appear to disassemble, leaving behind dense actin networks. After 1 h, we find condensates formed in the presence of actin and Arp2/3 to be of a smaller size on average (0.4 ± 0.1 μm in radius) compared to those formed without actin and Arp2/3 (1.2 ± 0.9 μm in radius) (Fig. 3*A*, *Upper* row). We conclude that actin filaments limit the growth of bulk N-WASP condensates. Next, we investigated whether surface condensation of *C. elegans* N-WASP on SLBs is impacted in a similar way by actin. We find that on SLBs condensates are smaller in the presence of actin and Arp2/3 (0.12 ± 0.04 μm in radius compared to 0.16 ± 0.06 μm without actin and Arp2/3) (Fig. 3*A*, *Lower* row and Movie S4). We observed similar effects of actin and Arp2/3 on human N-WASP surface condensates (*SI Appendix*, Fig. S5*B*). We conclude that N-WASP condensates concentrate actin monomers and serve as nucleation hubs for actin polymerization. Actin filaments limit N-WASP condensate size and even cause some of the N-WASP condensates to disassemble. Taken together, and similar to what is seen in the cortical condensates in the *C. elegans* zygote (25), our results are indicative of negative feedback of actin filaments onto N-WASP. This limits the amount of N-WASP in N-WASP surface condensates.

### Actin Polymerization Feeds Back on the Growth of N-WASP Condensates in an ATP-Independent Manner.

We next set out to understand whether the negative feedback mechanism of actin on N-WASP condensate growth is mediated by monomeric actin, filamentous actin, or if it requires a branched network (25). To answer this question, we investigated N-WASP bulk condensation in the presence of a polymerization inhibiting drug and with as well as without Arp2/3. We find that suppressing actin polymerization in the presence of Arp2/3 by addition of latrunculin A gives rise to bulk condensates that are still enriched with actin but that have a size (1.4 ± 0.9 μm in radius) that is not significantly different from the condition where latrunculin A is absent (1.2 ± 0.9 μm in radius, see above; Fig. 3*B*). N-WASP condensates still nucleate actin filaments in the absence of Arp2/3, and the resultant N-WASP condensates are slightly larger (0.6 ± 0.2 μm in radius) than in the presence of Arp2/3 (0.4 ± 0.1 μm in radius, see above; Fig. 3*B*). Together, this is consistent with Arp2/3-mediated negative feedback onto N-WASP, similar to what is seen in vivo (25). We conclude that this effect is due to the formation of branched actin networks. However, we cannot rule out other effects that are associated with Arp2/3, for example, an altered rate of actin polymerization, that could also enact the feedback.

Is this negative feedback dependent on ATP consumption? While actin polymerization is an active process, not all steps require ATP (34, 35). Filament elongation is ATP independent, and ATP molecules subsequently undergo hydrolysis within the actin filament (36). Likewise, ATP bound to Arp2/3 is hydrolyzed following filament branching (37). We next evaluated whether the size-limiting effect of polymerized actin on N-WASP condensates depends on ATP hydrolysis. To this end, we analyzed the growth of actin bound with the nonhydrolyzing ATP variant AMP-PNP in ATP-free buffer, and determined the effect on N-WASP condensation. We find that the kinetics of actin polymerization of nucleotide-exchanged actin are slowed down but do not completely stall (*SI Appendix*, Fig. S6). The final size of N-WASP condensates in the presence of nucleotide-exchanged actin and Arp2/3 in ATP-free buffer (0.4 ± 0.2 μm in radius) is similar compared to the ATP-rich condition (0.4 ± 0.1 μm in radius, see above; Fig. 3*B*). We conclude that N-WASP condensate size is limited by the N-WASP-dependent polymerization of actin filaments in a manner that is independent of ATP.

### Actin Polymerization Counteracts the N-WASP Prewetting Transition.

**Fig. 4.**
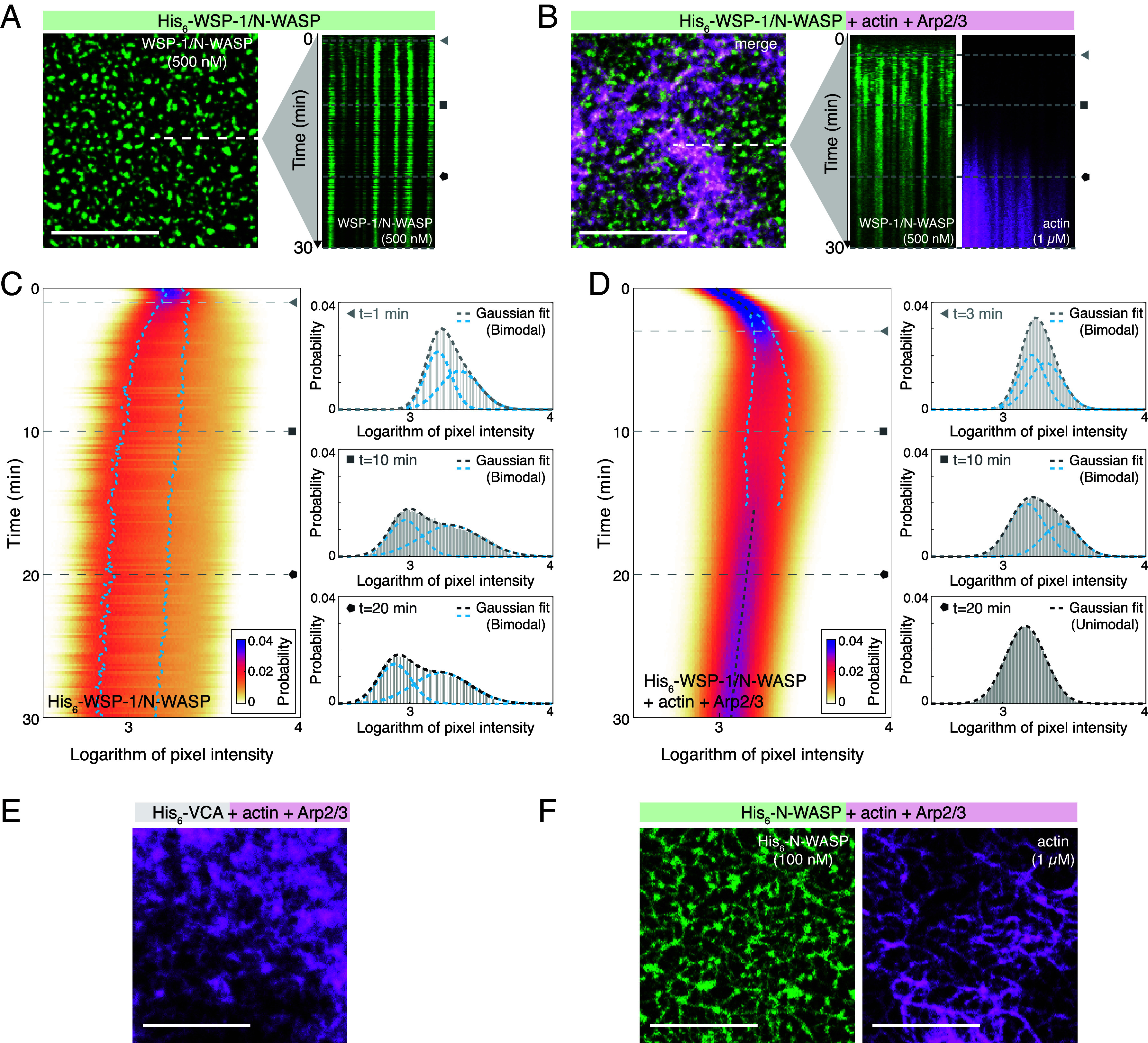
Actin polymerization counteracts prewetting. (*A*) Confocal images of binary mixture of His_6_-WSP-1 and His_6_-N-WASP (500 nM, 10% AF488 labeled, green) alone, and (*B*) in presence of actin (1 μM, 10% AF647 labeled, magenta) and Arp2/3 (10 nM) on SLBs after 30 min incubation. Kymographs along dotted line from 0 to 10 min (Movies S6 and S7 and *SI Appendix*, Fig. S15). (*C*) *Left*: Probability density kymographs of logarithm of N-WASP/WSP-1 pixel intensities for time-lapse images from (*A*). Gray lines (single Gaussian) and blue lines (sum-of-two-Gaussians) represent overlays from either Gaussian-fitting method, determined for each time point by comparing the fitting residues between the two methods. *Right*: Histograms at 3 distinct time points (marked with triangle, square, and pentagon) with either a single Gaussian or a sum-of-two-Gaussians fit (gray dashed lines). Blue dashed lines show respectively the fitted lower and higher Gaussian peaks in the case of a sum-of-two-Gaussians fits. (*D*) Same as in (*C*), Probability density kymographs of logarithm of N-WASP/WSP-1 pixel intensities for time-lapse images from the data with actin and Arp2/3 (*B*). (*E*) Confocal images of actin network (1 μM, 10% AF647 labeled, magenta) formed for 30 min on SLB with Arp2/3 (10 nM) and 100 nM His_6_-VCA or (*F*) full-length His_6_-N-WASP (green). (Scale bars: 10 μM.)

Actin nucleation via N-WASP and Arp2/3 is a major biochemical process for driving actin cortex formation (31, 38). We have observed both in vivo (25) and in vitro (Fig. 3*A*) that actin polymerization counteracts N-WASP condensation. We next ask whether actin polymerization impacts on the N-WASP prewetting transition. To ensure that N-WASP prewetting is approximately concomitant with actin polymerization, we used a binary mixture of *C. elegans* (250 nM) and human N-WASP (250 nM). Both variants condense on the supported lipid bilayers and colocalize (*SI Appendix*, Fig. S12). We compared the condensation kinetics of this N-WASP mixture alone (Fig. 4*A* and Movie S6) with the condensation kinetics in the presence of actin monomers and Arp2/3 complex (Fig. 4*B* and Movie S7). Similar to before (Fig. 2*C*) and as a hallmark of the prewetting transition, we observed in both conditions a split-up of the intensity histogram into a dilute and a condensed branch (Fig. 4 *C* and *D*). In the presence of actin and Arp2/3, the time of histogram splitting is commensurate with the stage of actin nucleation (*SI Appendix*, Fig. S13). Interestingly, while the split is maintained without actin and Arp2/3, it is reversed at a later time in their presence (Fig. 4*D* and *SI Appendix*, Fig. S13). This is indicative of an absence of N-WASP prewetting condensates at later times when actin filaments are present (*SI Appendix*, Figs. S14 and S15 and Movie S4). We observed similar but weaker effects when using human N-WASP instead of the binary mixture (*SI Appendix*, Fig. S16). Together, these results are consistent with actin polymerization, initiated in the condensates, suppressing the prewetting transition of N-WASP on lipid bilayers.

### Altering Cortex Architecture.

An in vivo actin cortex can display a complex architecture (39), and actin cortex structure is important for its functionality (40, 41). Can the nucleation of actin filaments from prewetting condensates alter the architecture of the resulting cortex, as compared to a homogeneously nucleated cortex? We formed in vitro actin cortices on supported lipid bilayers (1% DGS-Ni-NTA) either in the presence of full-length human N-WASP (100 nM) capable of forming prewetting condensates, or in the presence of the functional N-WASP domain VCA only (100 nM), which does not contain an intrinsically disordered domain and does not form condensates (42). We find that the presence of VCA leads to the formation of dense actin meshworks on the bilayer (Fig. 4*E*). However, cortices formed in the presence of N-WASP appear to branch more sparsely, showing a significantly higher degree of bundling. They more closely resemble an in vivo actomyosin cortex (Fig. 4*F*) (43). We conclude that nucleating actin filaments from N-WASP prewetting condensates results in an altered cortex architecture.

## Discussion

Protein condensates can form inside cells at specific times and locations, to support e.g. specific chemical reactions (3, 44). How exactly the formation and dissolution of condensates is regulated remains unclear in many instances. Here, we demonstrate that N-WASP at physiological concentrations can undergo a prewetting transition on lipid membranes, giving rise to N-WASP condensates that can drive actin polymerization reactions.

Prewetting transitions fall into the general class of wetting phenomena. Several examples of wetting phenomena have been observed in a biological context. Wetting of biological surfaces by protein condensates has been observed, for example, for transcription factors on DNA (9), for proteins on lipid membranes (45) and for proteins on microtubules (46). For the latter, TPX2 protein can wet microtubules and form a coat. Interestingly, this wetted layer has been shown to undergo a Rayleigh–Plateau instability, which drives the formation of evenly spaced droplets (47). In wetting phenomena, both the surface affinity and interfacial tension play a role in determining the condensate-surface contact morphology. The prewetting transition, on the other hand, is driven solely by the affinity between protein molecules and biological surfaces. As such, the presence of surfaces promotes the appearance of the condensed phase at bulk concentrations that are below the critical concentration for bulk phase separation (9). In this paper, we report evidence that N-WASP undergoes a prewetting transition on lipid membranes, forming N-WASP condensates that are limited to the bilayer. These can drive the nucleation of actin filaments, giving rise to branched actin networks with a more bundled architecture as compared to networks that are formed in the absence of N-WASP surface condensation [similar to other phase-separation induced actin network structures (21, 22)]. A key finding of the work here is that the growing actin meshwork itself appears to reverse the N-WASP prewetting transition. This is consistent with N-WASP reentering the dilute phase, a key aspect of the dynamic instability of cortical condensates in vivo (25). We speculate that counteracting prewetting plays an important role in orchestrating the switch from growth to shrinkage of cortical condensates in vivo, which suppresses coarsening for collectively maintaining an emulsified state (25). Future work will be required to understand the precise molecular mechanisms that underlie this actin-driven suppression of N-WASP prewetting. One possibility is that the actin filaments that grow from N-WASP surface condensates provide new possibilities for N-WASP association, effectively reducing the pool of N-WASP that is available for phase separation. In addition, the fact that bulk condensates are slightly smaller in the presence of Arp2/3 than they are without, could hint at a possible role of Arp2/3 in this negative feedback mechanism. Perhaps, the effect is imparted by N-WASP molecules associating to Arp2/3, again effectively reducing the pool of N-WASP that is available for phase separation.

Taken together, we suggest that N-WASP prewetting and its suppression by actin is important for the shaping of a functional actomyosin cortex architecture. Given the observed kinetics of N-WASP and actin condensates in *C. elegans* oocytes (25), it will be of interest to explore the potential role of prewetting for other processes of condensate formation in vivo.

## Materials and Methods

Additional details of experiments and analyses are described in *SI Appendix*, which includes *SI Appendix*, Figs. S1–S18 and Movies S1–S8 and *SI Appendix*, Table S1.

### Protein Expression and Purification.

N-WASP proteins were expressed in Sf9 cells using the baculovirus system (48). Untagged *C. elegans* WSP1 (UniProt Q17795, plasmid TH1828) was purified following a protocol for N-WASP purification of Ho et al. (49) (*SI Appendix*, Fig. 1*A*). MBP-His_6_-WSP1 (plasmid TH2035), His_6_-MBP-mGFP-WSP1 (plasmid TH1614), and MBP-His_6_-N-WASP (human, UniProt O00401, plasmid TH2098) were purified by His- and MBP-affinity columns and subsequent size-exclusion chromatography and stored in SEC buffer (50 mM HEPES (pH 7.4), 500 mM KCl, 5% glycerol, 1 mM DTT) as detailed in *SI Appendix*. For MBP-His_6_-WSP1 and MBP-His_6_-N-WASP the MBP moiety was cleaved off right before an experiment with 5% (v/v) GST-3C (in-house; 1 U/μl) for at least 30 min at room temperature. For His_6_-MBP-mGFP-WSP1 the MBP moiety was cleaved off with 5% (v/v) TEV protease (in-house; 1 U/μl) (*SI Appendix*, Fig. S1*B*). The samples were filtered with 0.1 μm PVDF centrifugal filters (UFC30VV, Merck) and the concentration was measured using adsorption at 280 nm and 488 nm on a Nano-Photometer (Implen, NP80). Actin was purified from rabbit muscle acetone powder following published protocols (50) and detailed in *SI Appendix*. Arp2/3 (from the porcine brain, #RP01P) and Cdc42 (his-tagged, human constitutively active mutant Q61L, #C6101-A) were purchased from Cytoskeleton Inc.

### Supported Lipid Bilayers.

SLBs were formed from small unilamellar vesicles (SUVs) composed of Synthetic 1-palmitoyl-2-oleoyl-glycero-3-phosphocholine (DOPC), 0 to 5% 1,2-dioleoyl-sn-glycero-3-[(N-(5-amino-1-carboxypentyl)iminodiacetic acid)succinyl] (nickel salt, DGS-NTA-Ni), 0.1% 1,2-dioleoyl-sn-glycero-3-phosphoethanolamine-N-[methoxy(polyethyleneglycol)-5,000] (ammonium salt) (PEG5000 PE) and 0.01% DiL (all Avanti Polar Lipids). Lipids were dissolved in chloroform, mixed, desiccated over night, and resuspended in SLB buffer (50 mM TRIS pH 7.5, 150 mM KCl, 1 mM DTT) at a final concentration of 1 mg/ml. SUVs were formed by 10× freeze–thaw cycles in liquid N_2_ and 37 ^°^C water bath, respectively, and subsequent extrusion through a 100 nm filter. SUVs were stored at −70 ^°^C and thawed and spun down at 17,000 × g for 45 min on the day of the experiment. For SLBs containing PIP2, 5% 18:0-20:4 PI(4,5)P2 were mixed with 95% DOPC and 0.1% PEG5000 in citrate buffer (150 mM KCl, 20 mM citrate, pH 4.8, 0.1 mM Ethylenediaminetetraacetic acid), extruded 3× through a 30 nm filter and used the same day. 96-well glass-bottomed plates (Greiner Bio one, CellView 655891) were washed with Hellmanex III (HÃ«lma Analytics) over night and rinsed under a stream of MilliQ H_2_O. Individual wells were washed with 6 M KOH for 2× 1 h, thoroughly rinsed with MilliQ H_2_O, and equilibrated with 35 μl SLB buffer. 15 μl SUVs were added per well and incubated for 5 min. SLBs were washed 5× with SLB buffer to remove excess SUVs. Buffer was exchanged right before the experiment to assay buffer and integrity of SLBs was checked by FRAP in each well. For experiments with Cdc42, 1 μM human His-Cdc42 was incubated 10 min prior to the assay on SLBs containing 0.1 to 5% DGS-NTA-Ni and unbound Cdc42 was washed away 3× with SLB buffer.

### Phase Separation Assays.

Freshly MBP-cleaved and filtered N-WASP proteins were prediluted to appropriate concentrations in SEC-buffer and 5 μl were mixed rapidly with 20 μl of actin polymerization buffer to obtain final concentrations of 15 mM Hepes, pH 7.4, 150 mM KCl, 0.1 mM MgCl_2_, and 0.2 mM ATP in low-binding eppendorf tubes by flickering. For experiments in the presence of actin, 5 μM N-WASP were mixed with actin polymerization buffer containing actin (3 μM final concentration, 5% Alexa-647-labeled) and Arp2/3 (100 nM final concentration) keeping the total volume and buffer conditions constant. As a control latrunculin A was added at a final concentration of 50 μM.

### Generation of Phase Diagrams.

To generate a phase diagram of the proteins we followed the method of Fritsch et al. (51). 20 μl of protein solution of three concentrations well above the critical point were transferred by pipetting with a cut tip into the wells of an ultralow attachment 384-well plate (CellCarrier 384 Ultra, Perkin Elmer, #6057800) in triplicates. The plate was spun down at 200×g for 10 min with a swinging-bucket rotor before transferring to the microscope. Confocal z-stacks of the whole well area were captured with a 20× air objective (Olympus U Plan XApo 20× (0.8 NA) Air) on a spinning disk confocal microscope (Olympus IXPLORE, IX83 inverted motorized stand with hardware autofocus and stage-top z-piezo). Analysis of the condensed volume versus the total volume was carried out in Matlab. The critical concentration c_sat_ is estimated by the axes intersection of the volume ratio of condensed protein (V_in_) over total volume (V_total_) versus bulk protein concentration. For the phase diagram of surface condensates on SLBs the condensed area fraction (A_in_ over total area A_total_ in the field of view) was measured by segmentation of condensates in Ilastik (see below). The critical concentration c_sat_ for surface condensation is estimated by the axes intersection of the condensed area fraction with the bulk protein concentration.

### Time-Lapse Imaging of In Vitro Condensates.

For time-lapse imaging of N-WASP condensation and actin polymerization, the buffer was equipped with a cocktail of triplet state quenchers following a protocol of Usaj et al. (52). Therefore, 10 ml of reaction buffer were prepared and mixed with trolox (freshly dissolved at 100 mM in methanol), cyclooctatetraene, and 4-nitrobenzyl alcohol (both from 200 mM stock in Dimethyl sulfoxide) to reach 2 mM final concentration of each component. To form trolox-quinone the solution was exposed to UV-light (254 nm, 10 min under a sterile hood) in a 10 cm petri dish on ice. Subsequently, solution was filtered (0.2 μm) and degassed (for 1 h in a desiccator on ice). As oxygen scavenger system, pyranose oxidase (1.4 mg/ml final concentration, NATE-1718, creative enzymes), catalase (0.01 mg/ml final concentration, C40), and 0.8% glucose were freshly mixed from frozen stocks and added to the reaction buffer 1 min before the start of the experiment. For ATP regeneration phosphocreatinine (10 mM, P7936) and creatine phosphokinase (53 U/ml, C3755) were added to the reaction mixture. Finally actin (10% AF647 labeled) and Arp2/3 were added to the buffer at the indicated concentrations and the mixture was added to the wells containing supported lipid bilayers. N-WASP was added into the wells by pipetting with a cut tip 5× up and down for mixing and imaging was started right after.

### Image Acquisition.

Confocal images and time-lapse movies were captured with a 100× Oil objective (Nikon, Plan Apochromat, DIC, 1.45 NA) on a spinning disk confocal microscope (Andor, inverted motorized Nikon stand with hardware autofocus) with 488, 561, and 647 laser lines. Time-lapse imaging was carried out in a single plane close to the cover glass and z-stacks were recorded after 1 h. Images were processed using Fiji/ImageJ 2.9.0. For size analysis of condensates in bulk, maximum projections were generated and condensates were segmented in Ilastik.

### FRAP Experiments.

FRAP experiments for condensates formed in bulk were carried out on droplets with a radius between 1.5 and 3.5 μm 5 min to 1 h after droplet formation. Photobleaching was performed for individual droplets using a 488 nm laser for 10× 50 μs over the full area of the droplet. Recovery of fluorescence intensity was recorded at a rate of 5 s/frame for 5 min. Data analysis was performed following the published protocol (29) and script of Lars Hubatsch, available here: https://gitlab.pks.mpg.de/mesoscopic-physics-of-life/DropletFRAP.

### Condensate Segmentation in Time-Lapse N-WASP Images.

Condensed fractions of N-WASP microscopy images were segmented using the Ilastik (53) software (version 1.4.0rc8). This neural network-based model was first trained with 1 to 2 representative snapshots from the image stack, then used to process the entire stack and generate segmentation results. For the kinetic assay of N-WASP surface condensation, the representative snapshot was taken as the first frame when condensates can be visually identified. In order to compare condensed fraction, a common model was trained from all representative snapshots (100 nM, 250 nM, and 500 nM bulk concentrations) and then used for time-lapse segmentation. For the FRAP assay of N-WASP condensates on lipid bilayers, the representative snapshots were taken as the frame before and subsequent to photobleaching. In order to reliably quantify FRAP intensity kinetics from dilute and condensed phases, tracking of individual condensates was performed and used to correct the segmentation results for frame-to-frame shifts. These segmentation results were finally exported from Ilastik and processed with custom Matlab scripts.

### Determination of Layer Index for N-WASP Surface Condensates.

The layer index quantification was developed as an indirect measurement to determine whether the observed surface condensates are close to being a single layer or multilayered. This quantification is enabled by the knowledge that the microscopy z resolution is on the same scale or larger than the typical thickness of N-WASP condensates (about 1μm observed from z-view). Therefore, fluorescent images acquired near the lipid bilayer are a close measurement of the volumetric fluorescence of surface condensates projected per area. For single-layer condensates, this volumetric fluorescence, integrated within the segmented condensate, shall scale linearly with the integrated condensate area ($V\propto A^{\alpha }$, α=1). For spherical condensates with a fixed contact angle instead, the volumetric fluorescence and area scale in a power law relation ($V\propto A^{\alpha }$, α=1.5). Thus, the value of such exponent α, which we name the layer index, serves as an indicator of condensate layers. To extract this index, pairs of integrated area and integrated intensity values were acquired from all segmented condensates, then a power law fit was performed.

### Quantification of N-WASP Prewetting Time.

To capture the abrupt appearance time $t_{\mathrm{pw}}$ of N-WASP condensates on lipid bilayers, two metrics were adopted: the condensate number increase per area quantified from the segmentation result, and the identification of condensed phase directly from N-WASP fluorescence intensities. In the first metric, the time of maximum condensate number increase per unit area was taken as the abrupt switch time. For the second metric, the logarithm of N-WASP fluorescence intensities (x) was first binned to calculate the probability distribution P(x,t) ($\sum_{x}P\left(x,t\right)=1$). A univariate and a two-variate (sum-of-two) Gaussian fit were then performed to generate the fitted probability density functions: $F_{1}\left(x,t\right)$ and $F_{2}\left(x,t\right)$ ($\int F_{i}\left(x,t\right)dx=1$). The residue between the fitted probability density function and the intensity data can be determined, $r_{i}\left(t\right)=\sum {\left[F_{i}\left(x,t\right)\Delta x-P_{i}\left(x,t\right)\right]}^{2}$, where Δx is the bin width. We note that compared to the two-variate Gaussian fit, a univariate fit from the same data distribution is bound to generate a larger residue value. Thus, an abrupt increase in the residue difference between the fit functions, $r_{0}\left(t\right)=r_{1}\left(t\right)-r_{2}\left(t\right)$, captures the abrupt appearance of the condensed N-WASP phase. The time of abrupt increase $t_{\mathrm{pw}}$ was therefore set as the time when this residue difference $r_{0}\left(t\right)$ increases above a common threshold (set to 1e-4 for N-WASP 100 nM, 250 nM, and 500 nM time series comparison and 2e-5 for WSP-1/N-WASP mix with or without actin comparison). Both metrics were first extracted per acquired image (time step: 5 to 10 s) and then spline-fitted to finer time steps (1 s). Finally, to offset the experimental time delay between bulk solution addition and start of image acquisition, we fitted the starting (0 to 30 s) kinetics of mean N-WASP fluorescent intensity to linear surface association dynamics. The time at which the mean intensity crossed zero was then extracted as the delay $t_{0}$ and was used in correcting for the switch time $t_{\mathrm{pw}}={\hat{t}}_{\mathrm{pw}}-t_{0}$ from both metrics. Error bars for the prewetting time identified via the Gaussian-fitting metric were determined from the SD across the four fields of view at each bulk concentration.

### Quantification of N-WASP Surface Concentrations at Prewetting.

To convert N-WASP fluorescence intensities $I_{s}\left(t\right)$ into a measurement of surface concentration $c_{s}\left(t\right)=\rho_{0}I_{s}\left(t\right)$, a rescaling factor $\rho_{0}$ is needed. To determine this rescaling factor, we note that the N-WASP surface association at low bulk concentrations closely resembles unsaturated first-order adsorption kinetics (*SI Appendix*). Thus, at the start of the surface association, N-WASP accumulation is approximately linear in time, $c_{s}\left(t\right)=k_{\mathrm{on}}c_{b}l\cdot t$, where $c_{b}$ is the applied bulk solution concentration and l the characteristic length for converting bulk to surface concentration units. $k_{\mathrm{on}}$, the association constant for N-WASP and lipid bilayers, stays a fixed number for datasets generated from the same experimental conditions (100 nM, 250 nM, and 500 nM N-WASP bulk concentrations) and can thus be normalized. The fact that the exact values of $k_{\mathrm{on}}$, $k_{\mathrm{off}}$ (disassociation constant), and characteristic length l remain unknown hinders the rescaling of the mean N-WASP intensity to true surface concentration units. Yet, $\rho_{0}$ can be determined such that for unsaturated adsorption (100 nM), the rescaled “surface concentration” $c_{s}\left(t\right)$ at long time is $c_{b}$ (100 A.U.). An extended discussion on the unit conversion and interpretation is included in *SI Appendix* and *Materials and Methods*. Finally, the prewetting time $t_{\mathrm{pw}}$ determined previously from the N-WASP condensed phase appearance was used to extract the rescaled N-WASP surface concentration ${\hat{c}}_{s}\left(t_{\mathrm{pw}}\right)=\rho_{0}I_{s}\left(t_{\mathrm{pw}}\right)$ at prewetting. Error bars for the surface concentrations identified via the Gaussian-fitting metric were determined from the SD across the four fields of view at each bulk concentration.

## Supplementary Material

Appendix 01 (PDF)

Movie S1. **WSP-1 condensates coarsen and fuse in bulk**. Confocal time-lapse imaging of *C. elegans* WSP-1 (5 μM, 10% 488-tagged, MBP-tag cleaved right before experiment and KCl concentration lowered to 150mM), in a plane close to the cover glass over the course of 150min. Field of view 20x20 *μ*m.

Movie S2. **WSP-1 condensates coarsen and fuse on supported lipid bilayers**. Confocal time-lapse imaging of *C. elegans* WSP-1 (100 nM, 10% 488-tagged, MBP-tag cleaved right before experiment) in actin polymerization buffer (containing 150mM KCl), in a plane close to the supported lipid bilayer (containing 1% Ni-NTA) over the course of 20min. Field of view 20x20 *μ*m.

Movie S3. **Actin polymerizes from bulk WSP1 condensates**. Confocal time-lapse imaging of *C. elegans* WSP-1 (5 *μ*M, 10% 488-tagged, green) mixed together with actin (3 *μ*M, 10% AF647 labeled, magenta) and Arp2/3 (100 nM) in actin polymerizing buffer containing 150mM KCl in a plane close to the cover glass over the course of 13.5min. Scale bar, 5 *μ*m.

Movie S4. **Actin polymerizes from WSP1 condensates on supported lipid bilayers**. Confocal time-lapse imaging of *C. elegans* WSP-1 (100 nM, 10% 488-tagged, green) mixed together with actin (1 *μ*M, 10% AF647 labeled, magenta) and Arp2/3 (100 nM) in actin polymerizing buffer containing 150mM KCl in a plane close to the supported lipid bilayer over the course of 20min. Scale bar, 5 *μ*m.

Movie S5. **N-WASP adsorption and condensation on supported lipid bilayers**. Confocal time-lapse imaging of human N-WASP (10% 488-tagged, MBP-tag cleaved right before experiment, upper row 100 nM, middle 250 nM, low 500 nM) in actin polymerization buffer (containing 150mM KCl), in a plane close to the supported lipid bilayer (containing 1%Ni-NTA) over the course of 10min. Scale bar, 5 *μ*m.

Movie S6. **N-WASP adsorption and condensation on supported lipid bilayers**. Confocal time-lapse imaging of binary mixture of human N-WASP and *C. elegans* WSP-1 (500 nM, 10% 488-tagged, MBP-tag cleaved right before experiment) in actin polymerization buffer (containing 150mM KCl), in a plane close to the supported lipid bilayer (containing 1%Ni-NTA) over the course of 30min. Scale bar, 5 *μ*m.

Movie S7. **Actin polymerizes from N-WASP/WSP-1 condensates on supported lipid bilayers**. Confocal time-lapse imaging of binary mixture of human N-WASP and *C. elegans* WSP-1 (500 nM, 10% 488-tagged, green) mixed together with actin (1 *μ*M, 10% AF647 labeled, magenta) and Arp2/3 (10 nM) in actin polymerizing buffer containing 150mM KCl in a plane close to the supported lipid bilayer over the course of 30min. Scale bar, 5 *μ*m.

Movie S8. **N-WASP forms clusters on supported lipid bilayers in the presence of Cdc42**. Confocal time-lapse imaging of *C. elegans* WSP-1 (100 nM, 10% 488-tagged) in actin polymerization buffer (containing 150mM KCl), in a plane close to the supported lipid bilayer (containing 1% Ni-NTA) over the course of 20min. The supported lipid bilayer was incubated with 1 *μ*M His-Cdc42 before the experiment. Field of view 20x20 *μ*m. Scale bar, 5 *μ*m.

## Data Availability

Microscopy images and custom codes are publicly available at https://doi.org/10.17617/3.FOX6GL (54).
