# Supplementary figures and images for: Actin polymerization counteracts prewetting of N-WASP on supported lipid bilayers
Source: Proc Natl Acad Sci U S A. 2024 Dec 4;121(50):e2407497121. doi: 10.1073/pnas.2407497121 (PMC11648614; doi:10.1073/pnas.2407497121)

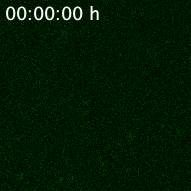

Supplement: Movie S1. — WSP-1 condensates coarsen and fuse in bulk. Confocal time-lapse imaging of C. elegans WSP-1 (5 μM, 10% 488-tagged, MBP-tag cleaved right before experiment and KCl concentration lowered to 150mM), in a plane close to the cover glass over the course of 150min. Field of view 20x20 μm. [file pnas.2407497121.sm01.gif]

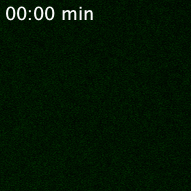

Supplement: Movie S2. — WSP-1 condensates coarsen and fuse on supported lipid bilayers. Confocal time-lapse imaging of C. elegans WSP-1 (100 nM, 10% 488-tagged, MBP-tag cleaved right before experiment) in actin polymerization buffer (containing 150mM KCl), in a plane close to the supported lipid bilayer (containing 1% Ni-NTA) over the course of 20min. Field of view 20x20 μm. [file pnas.2407497121.sm02.gif]

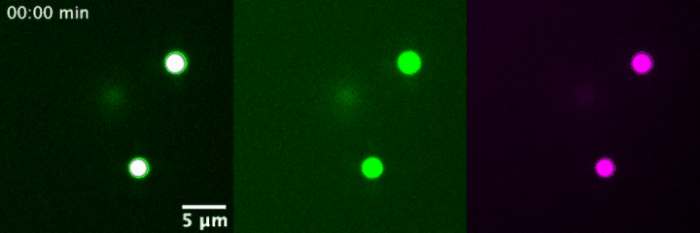

Supplement: Movie S3. — Actin polymerizes from bulk WSP1 condensates. Confocal time-lapse imaging of C. elegans WSP-1 (5 μM, 10% 488-tagged, green) mixed together with actin (3 μM, 10% AF647 labeled, magenta) and Arp2/3 (100 nM) in actin polymerizing buffer containing 150mM KCl in a plane close to the cover glass over the course of 13.5min. Scale bar, 5 μm. [file pnas.2407497121.sm03.gif]

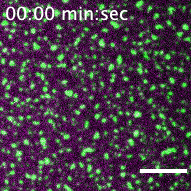

Supplement: Movie S4. — Actin polymerizes from WSP1 condensates on supported lipid bilayers. Confocal time-lapse imaging of C. elegans WSP-1 (100 nM, 10% 488-tagged, green) mixed together with actin (1 μM, 10% AF647 labeled, magenta) and Arp2/3 (100 nM) in actin polymerizing buffer containing 150mM KCl in a plane close to the supported lipid bilayer over the course of 20min. Scale bar, 5 μm. [file pnas.2407497121.sm04.gif]

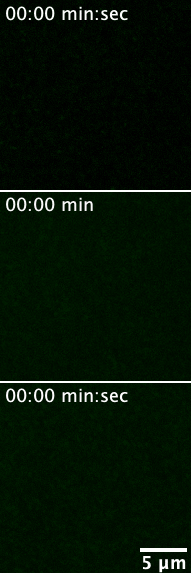

Supplement: Movie S5. — N-WASP adsorption and condensation on supported lipid bilayers. Confocal time-lapse imaging of human N-WASP (10% 488-tagged, MBP-tag cleaved right before experiment, upper row 100 nM, middle 250 nM, low 500 nM) in actin polymerization buffer (containing 150mM KCl), in a plane close to the supported lipid bilayer (containing 1%Ni-NTA) over the course of 10min. Scale bar, 5 μm. [file pnas.2407497121.sm05.gif]

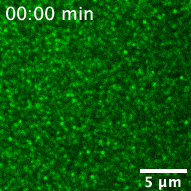

Supplement: Movie S6. — N-WASP adsorption and condensation on supported lipid bilayers. Confocal time-lapse imaging of binary mixture of human N-WASP and C. elegans WSP-1 (500 nM, 10% 488-tagged, MBP-tag cleaved right before experiment) in actin polymerization buffer (containing 150mM KCl), in a plane close to the supported lipid bilayer (containing 1%Ni-NTA) over the course of 30min. Scale bar, 5 μm. [file pnas.2407497121.sm06.gif]

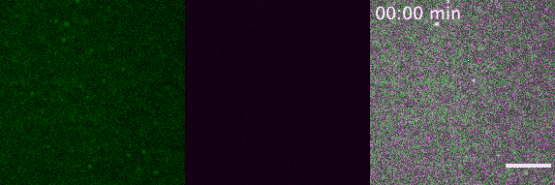

Supplement: Movie S7. — Actin polymerizes from N-WASP/WSP-1 condensates on supported lipid bilayers. Confocal time-lapse imaging of binary mixture of human N-WASP and C. elegans WSP-1 (500 nM, 10% 488-tagged, green) mixed together with actin (1 μM, 10% AF647 labeled, magenta) and Arp2/3 (10 nM) in actin polymerizing buffer containing 150mM KCl in a plane close to the supported lipid bilayer over the course of 30min. Scale bar, 5 μm. [file pnas.2407497121.sm07.gif]

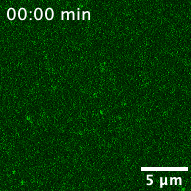

Supplement: Movie S8. — N-WASP forms clusters on supported lipid bilayers in the presence of Cdc42. Confocal time-lapse imaging of C. elegans WSP-1 (100 nM, 10% 488-tagged) in actin polymerization buffer (containing 150mM KCl), in a plane close to the supported lipid bilayer (containing 1% Ni-NTA) over the course of 20min. The supported lipid bilayer was incubated with 1 μM His-Cdc42 before the experiment. Field of view 20x20 μm. Scale bar, 5 μm. [file pnas.2407497121.sm08.gif]
